# Periosteal nerve blocks for distal radius and ulna fracture manipulation—the technique and early results

**DOI:** 10.1186/s13018-015-0277-6

**Published:** 2015-09-02

**Authors:** M. Elhosseini Tageldin, Mamun Alrashid, Al-Achraf Khoriati, Srinivas Gadikoppula, Henry Dushan Atkinson

**Affiliations:** Department of Trauma and Orthopaedics, North Middlesex University Hospital, London, N18 1QX UK; St George’s Hospital, Blackshaw Rd, Tooting, London, SW17 0QT UK

## Abstract

**Background:**

We present a pilot series of patients with distal forearm fractures manipulated following a proximal periosteal nerve block with local anaesthesia.

This is a novel technique which can be utilised in adults and children and is described herein.

**Methods:**

With a median of 40 years (range 10–81 years), 42 patients (16 children) with distal radial and ulnar fractures were included. Of these patients, 40 underwent periosteal blocks in the emergency room or fracture clinic; 2 were already inpatients. Fractures were manipulated routinely and immobilised with plaster. Mobile fluoroscopy was not used for patients in the emergency department or fracture clinic.

**Results:**

Of the 42 patients, 40 patients (95 %) had successful fracture manipulation and did not require subsequent treatment. Two patients (5 %) needed subsequent surgery, one for K-wire stabilisation of their fracture and the second for volar plate fixation. The procedure was described as painless in 35 (83 %) patients (visual analogue scale/VAS score 0), with 6 (14 %) suffering minimal pain (VAS 1–3). In the 12–16-year age group, 15 patients (94 %) described the manipulation as painless; 1 patient described the procedure as minimally painful. No additional analgesia of any kind was given. There were no direct complications from any of the periosteal nerve blocks.

**Conclusions:**

Local anaesthetic periosteal nerve blocks injected proximally to the fracture sites are a simple and yet very effective new technique which provide good/excellent analgesia and facilitate the reduction of distal radial and ulnar fractures.

## Introduction

Distal radius and ulna fractures are some of the most common bony injuries [1], with a reported UK annual adult incidence of 9/10,000 men, 37/10,000 women [2] and 16/1000 children [3]. In adults, these fractures are usually treated in an outpatient setting; however, around 20 % (mainly elderly patients) require inpatient hospital management [2].

The key to effectively managing these patients lies with the initial fracture manipulation. By obtaining adequate analgesia, one can manipulate the majority of these injuries and avoid the need for costly surgery and hospital admissions. Various regional anaesthetic techniques are commonly used including haematoma block, intravenous regional anaesthesia (Biers block), sedation and brachial plexus block; the first three of these techniques are frequently used in the emergency room setting [4].

Biers block has been shown to be more effective than haematoma block in one series and resulted in a lower fracture remanipulation rate (4/72 vs. 17/70 fractures, respectively), superior post-manipulation radiographs and less reported pain [5]. However, there is a risk of local anaesthetic leakage into the circulation with both the Biers and haematoma blocks. This can lead to cardiac arrhythmias, hypotension [6] and central nervous system effects including tinnitus, dizziness, drowsiness and convulsions [7]. Sedation using a combination of benzodiazepines and opioid analgesia should be used with caution, particularly in elderly patients with multiple medical comorbidities, and can often lead to hospital admission for patient monitoring [8]. Brachial plexus blocks also need hospital admission and require anaesthetic expertise and so are rarely used. A Cochrane review of all these techniques did not find any superiority of one method over another [9].

The proximal periosteal block was conceived when the lead author was once faced with a clinical scenario in the emergency department where an 11-year-old boy had sustained an angulated greenstick fracture of the distal radius and ulna. The parents had refused to allow a hospital admission, so a 1 % lidocaine solution was injected around the radius and ulna, about 4 cm proximal to the fracture angulation site. Following this, a painless manipulation was performed, successfully restoring the bony alignment. Basically, there is almost no haematoma in greenstick fractures, and it was concluded that the local anaesthetic had most probably created an effective periosteal nerve block.

Bones have a complex autonomic and sensory nerve supply. The nerves accompany the nutrient arteries in the perivascular spaces within the Haversian systems and supply osteocytes with polypeptides that help to regulate osteoclastic and osteoblastic activity [10]. However, there is still no agreement about the exact anatomical peripheral afferent pathways of the sensory pain fibres from the pain receptors in the periosteum.

Following this initial observation, this pilot series was conceived in order to evaluate the efficacy of this technique of administering local anaesthesia proximal to the fracture (not into the fracture haematoma). To our knowledge, this technique has not been previously described.

## Patients and methods

The series included patients aged 10 years and older with displaced, closed distal radial fractures (with or without associated distal ulnar fractures) which required a manipulation either as the definitive treatment or in order to achieve a preliminary reduction in grossly displaced intra-articular fractures which would require subsequent operative fixation. We excluded multiple injured or patients unconscious from head injuries, patients with open fractures, children under 10 years of age and patients refusing local anaesthesia. Though no patients we encountered during to study had any clinical evidence of a compartment syndrome in the same limb, this too would have been considered an exclusion criterion. The study was carried out in accordance with the Helsinki Declaration and was given Research Ethics Committee approval by the NRES Committee London, with an REC reference 11/LO/1411.

The series consisted of 42 consecutive patients (31 females) managed by one orthopaedic team over a period of 6 months. Median patient age was 40 years (range 10–81 years). Fractures were classified according to Frykman system (Table 1).

**Table 1:** Distribution and type of wrist fracture

Frykman classification	Type/number of patients	Type/number of patients (with ulnar fracture)
Extra-articular radial fracture	I/15	II/2
Intra-articular fracture involving radio-carpal joint	III/10	IV/9
Fracture of radio-ulnar joint	V/1	VI/0
Intra-articular involving radio-carpal and radio-ulnar joints	VII/2	VIII/3

All 42 patients underwent proximal periosteal blocks using the described technique. Informed consent was obtained from all the patients. The procedure was explained in full to the children and their parents, and all the children were willing participants. Patients were asked to grade their levels of pain using a visual analogue score (VAS) pain scale (0–10 ranging from no pain up to severe pain).

When considering performing a wrist block, one should note that the terminal sensory branch of the radial nerve lies close to the lateral side of the radial artery in the middle third of the forearm [11]. The nerve leaves the artery 7 cm proximal to the wrist joint to curve around the lateral side of the radius, piercing the deep fascia on the dorsal aspect. Thus, there is little risk of injuring the nerve when injecting a periosteal block around 6–8 cm proximal to the wrist joint. By injecting circumferentially around the radius and ulna, close to the bones, there is little risk of injuring the radial and ulnar arteries and nerves.

**Fig. 1:**
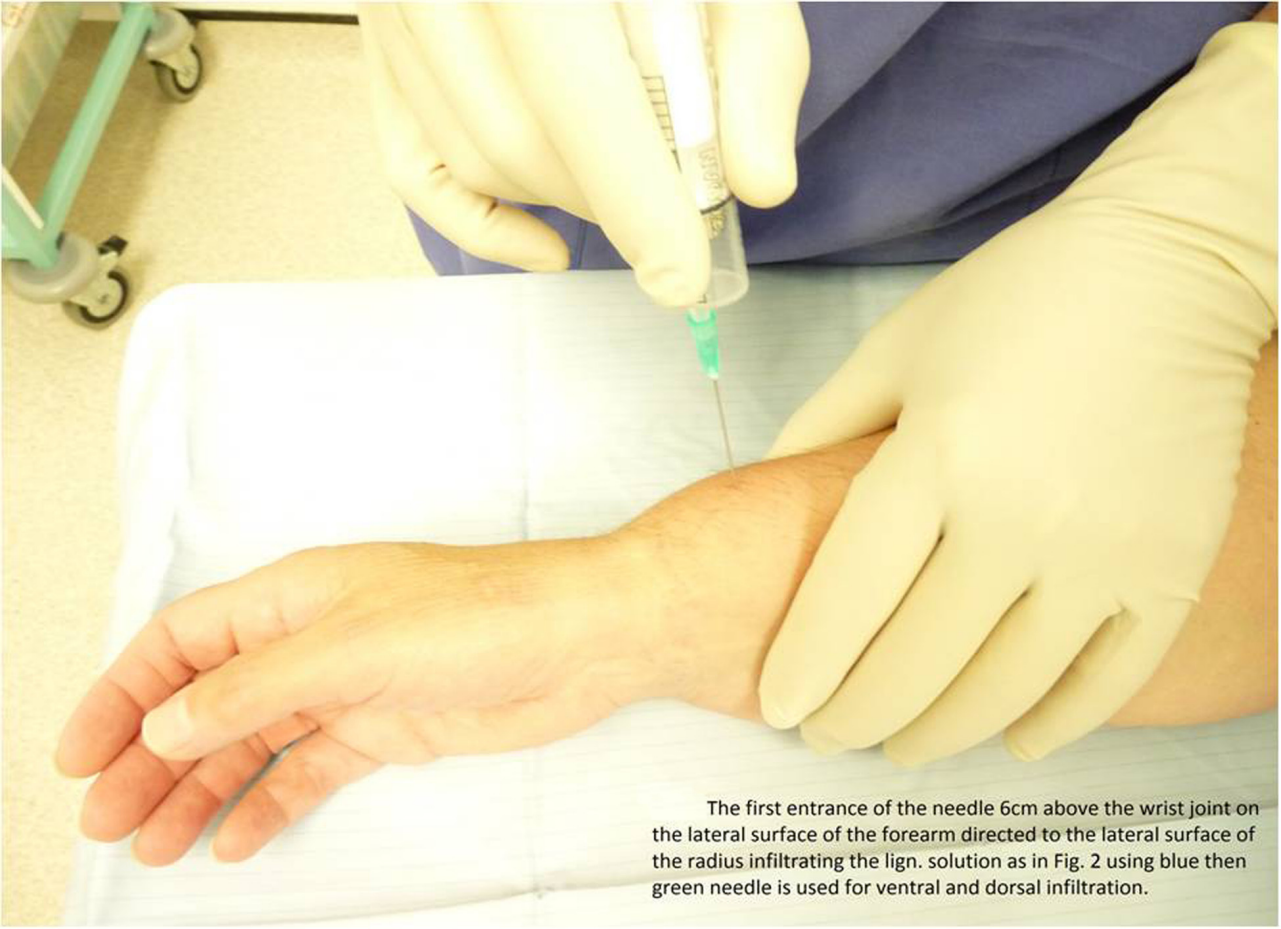
Following an initial injection of local anaesthetic to anaesthetise the skin on the lateral surface of radius, a second injection provides circumferential anaesthesia around the radius and ulna (around 6 cm proximal to wrist joint)

The patient is positioned supine on a trolley or procedure table with their hand and forearm resting on a dressing table (Fig. 1), preferably supported by a pillow. A precautionary intravenous cannula is inserted in the uninjured arm. Oxygen and intravenous fluids must be available, as is routine with all regional anaesthesia (though these were not required in any of our cases).

The injured forearm is typically infiltrated under aseptic conditions with 10–15 ml of 1 % lidocaine in adults and around 0.2–0.3 ml of 1 % lidocaine per kg of body weight (not exceeding 3 mg per kg of body weight in children). It should be done preferably in quiet surroundings, such as in the emergency treatment room/theatre, with the clinician seated.

**Fig. 2:**
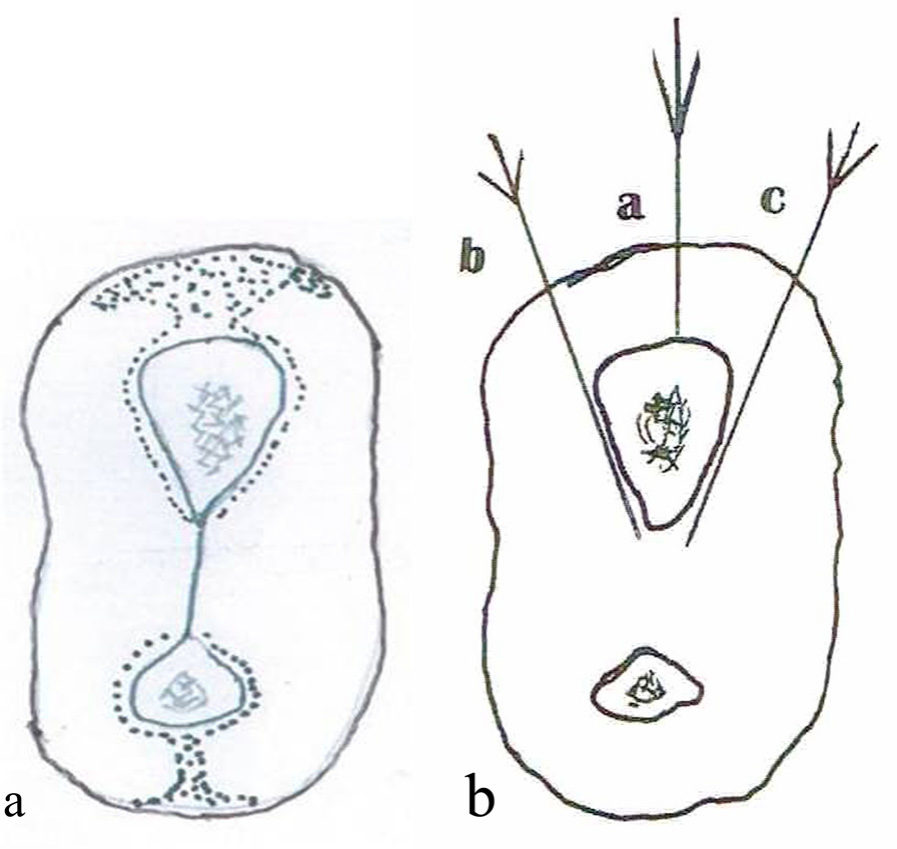
**a** A schematic cross section of the distal forearm 6 cm proximal to the wrist joint. The *dotted zones* indicate the sites for lidocaine solution infiltration. **b** The directions of the needle abutting the lateral/radial, ventral and dorsal surfaces of radius

The injection is started on the lateral/radial side. Using a 10-ml syringe with an orange or blue needle, the needle enters just behind the cephalic vein, 6 cm proximal to wrist joint, infiltrating the subcutaneous tissue in an area across the lateral surface of the radius about 1 × 2 cm. Next, the needle is directed at a right angle/perpendicular to the radius until the needle tip abuts the lateral aspect of the radius as shown in Fig. 2a, b.

Lidocaine is injected centrally (towards the radius itself) and slightly anteriorly (ventrally) and posteriorly (dorsally) to cover the whole lateral surface of radius. The needle is then withdrawn and replaced by a green needle and inserted using the same entry point. It is easier to start with the ventral surface. Before advancing the needle any further, one can roll the skin/soft tissue downwards and away from the needle at that level (using the clinician’s thumb), such that the needle is more easily directed towards the outermost part of the ventral surface. The needle is then advanced to touch the bone, and the injection is continued across the radius inserting approximately 0.5 ml of lidocaine solution for every 0.5 cm needle advancement, with the needle touching the bone, and continuing until the bone can no longer be felt with the needle tip. The needle is then withdrawn. The skin is then rotated/rolled posteriorly, and using the same entry point, the needle is directed towards the dorsal surface and continued as described for the ventral side. In a large wrist, one might need a second dorsal entry point to allow for the rounded contour of the dorsal surface.

**Fig. 3:**
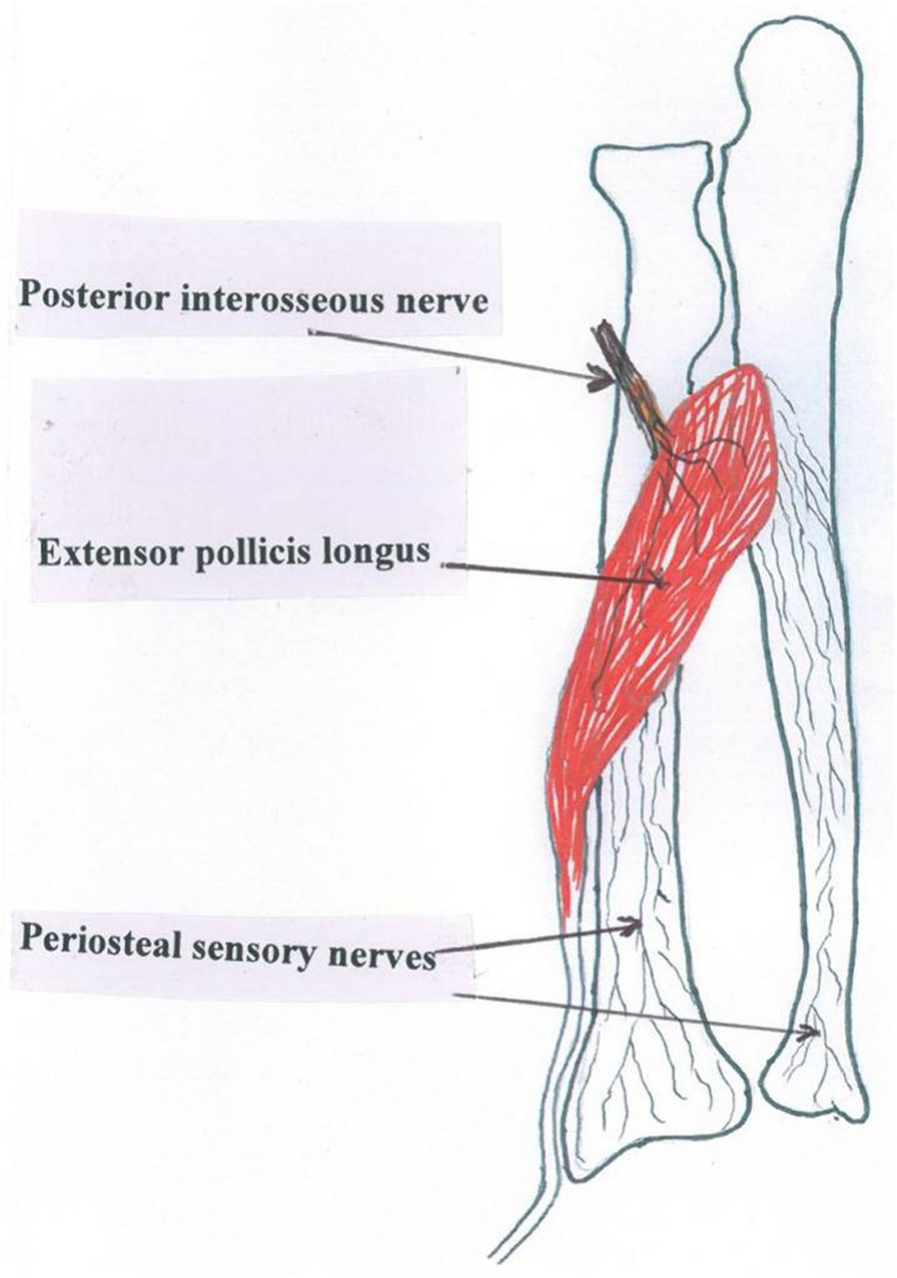
A schematic of the forearm showing the sensory nerve network from the posterior interosseous nerve flowing via the extensor pollicis longus muscle

The process is repeated for the ulna with approximately 3 ml of lidocaine solution if there is a concurrent ulnar fracture (regardless of whether or not the ulnar fracture is displaced). Figure 3 shows the periosteal nerve supply.

Fifteen minutes after the injections, a manipulation is performed and the wrist is then supported in either a below elbow or above elbow backslab or full plaster cast, in the same way one would normally manage these injuries. The whole procedure takes between 45 and 50 min. Post-manipulation radiographs are then obtained.

## Results

Of the 42 patients, 37 patients were manipulated under periosteal block in the emergency department and went home the same day. The manipulations typically took place between 3 and 4 h following injury. Three patients were treated in the fracture clinic, having previously had inadequate reductions in the emergency department under a haematoma block. The remaining two patients were already inpatients in the hospital, one was medically unfit for surgery and the second had been admitted for a remanipulation of a 1-week-old distal radial fracture under general anaesthesia, but surgery had been delayed. Both patients were also manipulated under proximal periosteal block.

Of the 42 patients, only 2 patients needed subsequent surgery, 1 for K-wire stabilisation of their fracture and the second for volar plate fixation. Forty patients (95 %) had successful manipulations and did not require further interventions.

**Fig. 5:**
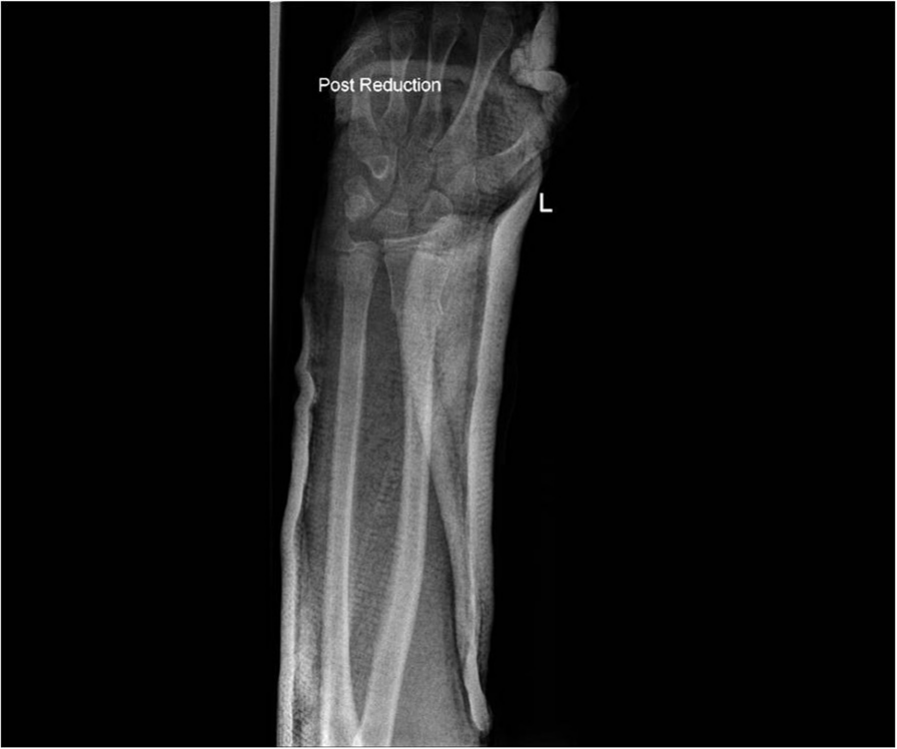
Pre- and post-manipulation radiographs of an 11-year-old boy with a left distal forearm fracture

**Fig. 6:**
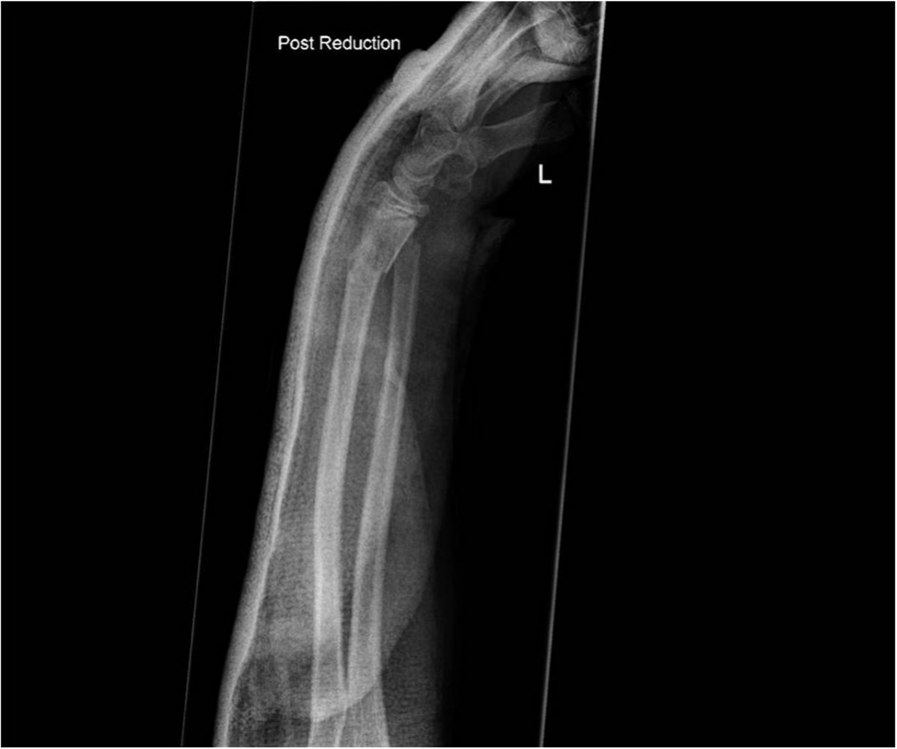
Pre- and post-manipulation radiographs of an 11-year-old boy with a left distal forearm fracture

**Fig. 7:**
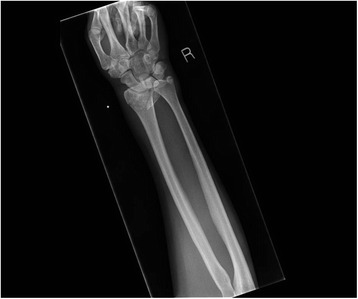
Pre- and post-manipulation radiographs of a 33-year-old lady with a right distal radius fracture

**Fig. 8:**
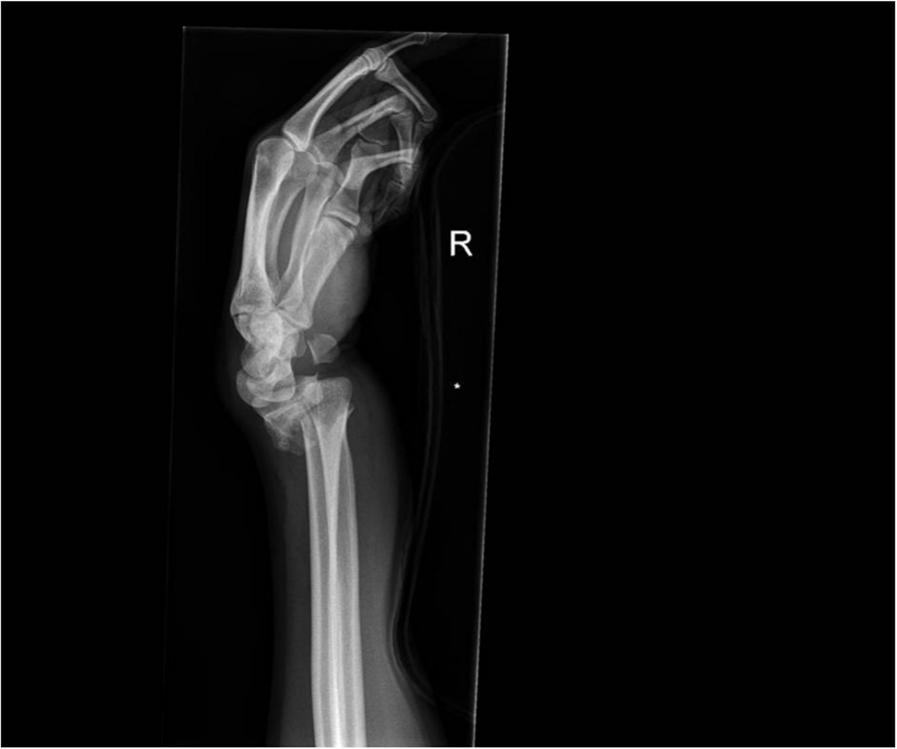
Pre- and post-manipulation radiographs of a 33-year-old lady with a right distal radius fracture

**Fig. 9:**
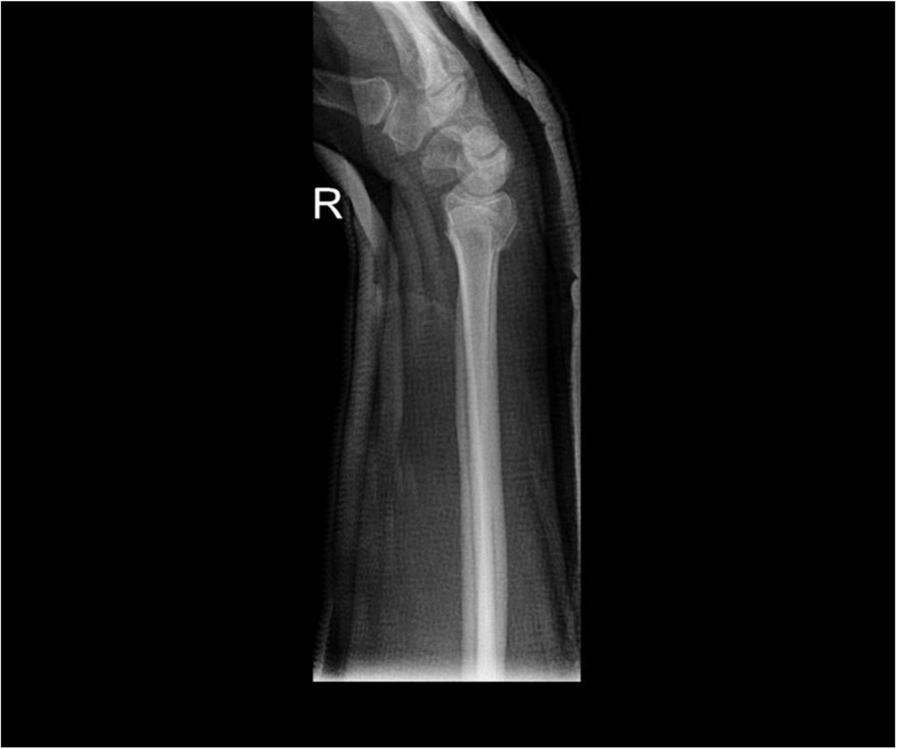
Pre- and post-manipulation radiographs of a 33-year-old lady with a right distal radius fracture

**Fig. 10:**
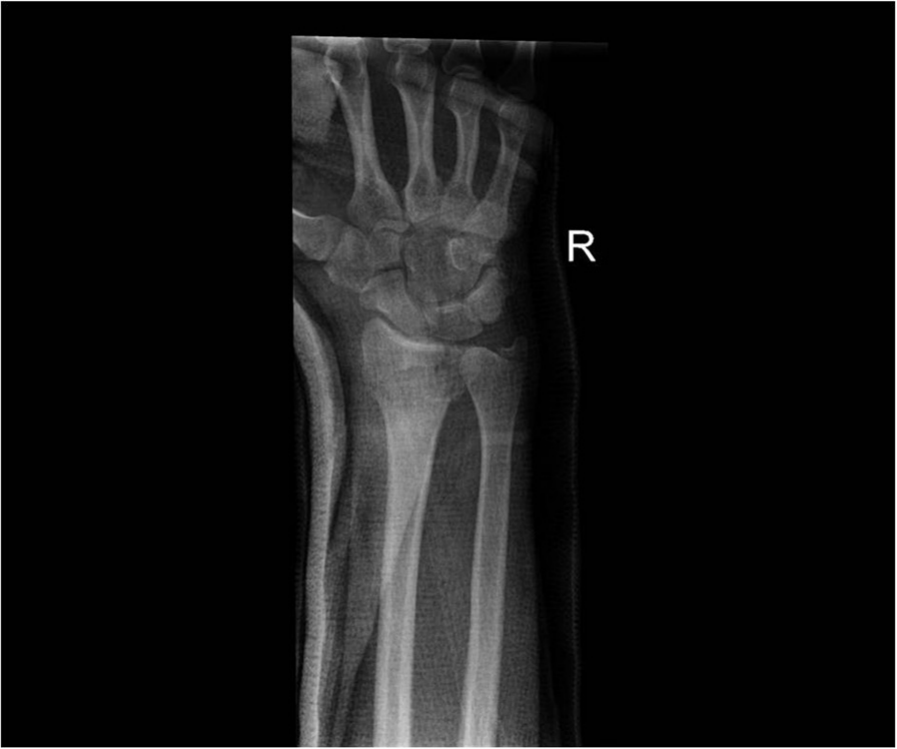
Pre- and post-manipulation radiographs of a 33-year-old lady with a right distal radius fracture

All patients were followed up to bony and clinical fracture union. Figures 4, 5, 6, 7, 8, 9 and 10 show radiographs of two patients.

**Fi g. 4:**
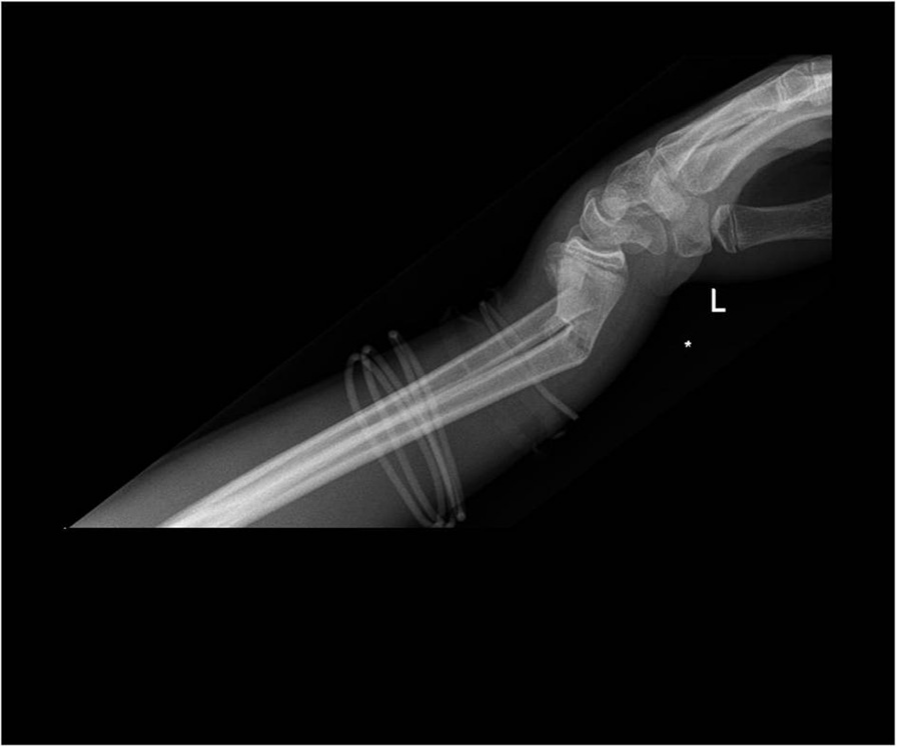
Pre- and post-manipulation radiographs of an 11-year-old boy with a left distal forearm fracture

The patients were asked to score their pain levels during the fracture manipulation. The procedure was described as painless in 36 (83 %) of patients (VAS score 0), with 14 % suffering only minimal pain (VAS 1–3). In the 12–16-year age group, 15 patients described the manipulation as painless. One described it as minimally painful (Table 2). No additional analgesia of any kind was given. There were no direct complications from any of the periosteal nerve blocks, and no other complications were noted.

**Table 2:** Grades of pain experienced by patients

Age groups	Total patients	Painless VAS 0	Minimal pain VAS 1–3	Painful VAS >3
12–16 years	16	15	1	Nil
16–50 years	11	8	3	Nil
>50 years	15	13	2	Nil

## Discussion

A previous study looking at the costs of managing distal radial fractures in the UK’s National Health Service estimated a figure of £320.50 (400 Euros, $500 USD) per patient in 1997 [12]; it is likely that this figure is now even greater. The bulk of this expenditure was due to patients requiring hospital admission, and it was concluded that if admission into hospital was not necessary, and that if the subsequent costs of the admission, the theatre operating time and staff costs were removed, then significant savings could be made. One of the primary reasons for hospital admission is to re-manipulate previously inadequately manipulated fractures. High re-manipulation rates are often the result of patients having had suboptimal analgesia during their initial manipulation.

This study found that the proximal periosteal block is a very acceptable method of facilitating distal forearm fracture manipulation. It provides an excellent mode of pain relief resulting in high patient satisfaction levels, low re-manipulation rates and does not require hospital admission. This is particularly the case in the 12–16-year age group. Only two patients in this series required further intervention. Both these fractures required internal fixation; and this was more due to the nature of the fracture pattern.

Indeed, it is completely acknowledged that some fracture patterns, particularly unstable and intra-articular fracture configurations, inevitably require surgical fixation, and a manipulation alone is not sufficient in these cases; and cultural differences in managing these injuries exist in different settings and countries. However, we would argue that this technique is still beneficial in the initial emergency management of even these more complex cases, particularly if their definitive management is likely to be delayed by several days.

Our sample was obtained over a 6-month period from patients presenting to a single emergency department during the on-call take of one orthopaedic team. Though small, this consecutive series of 42 patients consisted mainly of adult females over 50 years of age, and adolescent boys, reflecting the typical bimodal distribution of these injuries. Most of the blocks were performed by the lead author; however, we found that there was only a very short learning curve, and the technique was quite reproducible by both senior and junior members of the team.

We recognise that there are limitations to this small study. There was no control group, and no comparisons were made with the results of other patients being managed in our hospital using the more traditional haematoma blocks. However, we have demonstrated the efficacy and great potential of this technique, and it is well known that additional analgesia is needed sometimes with haematoma block. Having run this pilot series, our intention is to now run a prospective randomised control trial comparing the proximal periosteal block to the haematoma block in order to further evaluate the technique.

## Conclusion

Local anaesthetic periosteal nerve blocks injected proximally to the fracture sites are a simple and yet very effective new technique which provide good/excellent analgesia and facilitate the reduction of distal radial and ulnar fractures.
